# Damage State Recognition and Quantification Method for Shield Machine Hob Based on Deep Forest

**DOI:** 10.3390/s26051586

**Published:** 2026-03-03

**Authors:** Huawei Wang, Qiang Gao, Sijin Liu, Peng Liu, Xiaotian Wang, Ye Tian

**Affiliations:** 1 China Railway 14th Bureau Group Co., Ltd., Jinan 250011, China; wanghuawei.14g@crcc.cn (H.W.); ahlsj@126.com (S.L.); liupeng202410@126.com (P.L.); ddgyjy_ty@163.com (Y.T.); 2 China Railway Construction Underwater Shield Tunnel Engineering Laboratory, Jinan 250011, China; 3 The Department of Mechanical Engineering, Dalian University of Technology, Dalian 116024, China; wangxiaotian@mail.dlut.edu.cn

**Keywords:** machine learning, deep forest, signal processing, shield machine hob, wear monitoring, hob damage

## Abstract

The damage status of shield machine disc cutters directly impacts the safety and efficiency of tunnelling projects. Current manual inspection methods involve high risks and low efficiency, while existing detection methods suffer from low accuracy and poor real-time performance in complex environments, often lacking quantitative analysis capabilities. To address these issues, this paper proposes an intelligent identification and quantitative assessment method for disc cutter damage based on the Deep Forest (DF) model. First, an eddy current sensor calibration platform was established, and a mapping relationship between output voltage and actual wear was developed through piecewise fitting to achieve precise wear quantification. In the data preprocessing stage, signal quality was improved via filtering, and typical damage features such as edge chipping, cracks, and eccentric wear were extracted using pulse edge detection. These feature segments were then resampled to construct the model training dataset. The DF model utilizes a hierarchical ensemble structure to mine data correlations, enabling accurate identification of four states: normal, edge chipping, eccentric wear, and cracks. Simultaneously, a DF regression model was employed to provide continuous quantitative predictions of damage size. Experimental results show that the classification model achieved accuracies of 98%, 96%, and 96% on the training, validation, and test sets, respectively, with weighted average F1-scores exceeding 0.96. The regression model achieved a coefficient of determination (R^2^) of 0.9940 and a root mean square error (RMSE) of 0.4051 on the test set. Both models demonstrate excellent performance and generalization, achieving full coverage from “qualitative state identification” to “quantitative wear assessment,” thereby providing reliable decision support for cutter maintenance and replacement.

## 1. Introduction

Slurry shield tunnelling is widely utilized in tunnel projects across complex, water-rich strata such as rivers and straits, where operational safety directly impacts project quality and construction duration [1,2]. As the core components responsible for rock breaking in the slurry shield cutterhead system, disc cutters maintain long-term contact with high-strength rock during excavation. Their wear evolution is decisive for propulsion performance, cutterhead stress status, and construction continuity [3,4].

In practical engineering, the working environment for slurry shields is extremely harsh [5]. Under complex geological conditions, abnormal wear forms—such as edge chipping, fracturing, and eccentric wear—account for a major proportion of unplanned downtime and cutterhead maintenance caused by cutter failure [6,7]. Therefore, achieving real-time, accurate monitoring and effective early warning of cutter damage, especially in the early stages of abnormal wear, is a key technical challenge for ensuring safe and efficient slurry shield excavation [8]. Currently, the assessment of disc cutter damage still relies heavily on manual hyperbaric inspections, a method characterized by high operational risks and low efficiency. To address the limitations of manual inspection, researchers globally have conducted extensive studies on cutter wear prediction and status identification, which can be categorized into three main approaches.

The first category is based on empirical models and statistical regression analysis, typically establishing empirical mapping relationships between wear volume and operating parameters using field data. For instance, Oraby S et al. utilized multivariate non-linear regression to model wear, tool life, and initial cutting conditions for wear prediction [9]. S M Amaitik et al. established tool life models using multivariate linear regression [10]. While applicable under specific conditions, these methods face limitations in prediction accuracy and real-time performance under complex geological and dynamic environments.

The second category is based on rock-breaking mechanics and wear theory, constructing prediction models from the perspective of cutter–rock interaction mechanisms. Lihui Wang et al. analyzed the wear mechanism of the cutter ring surface and proposed a new theoretical model for tool wear evolution [11]. She Lei et al. derived wear rate and life indicators by introducing the calibration expression of the normal load from the CSM model, establishing a theoretical wear prediction model [12]. Dong Shen et al. utilized Abaqus to simulate the stress state of cutters during rock fragmentation and, based on the abrasive wear mechanism, derived radial wear and linear wear rate prediction models for single disc cutters [13]. Lihui Wang et al. further established a model to predict wear evolution by analyzing the internal relationships between cutter motion, loads, and rock frictional properties [14]. Although these methods offer strong physical interpretability, they are highly sensitive to geological parameters and complex operating conditions, making their stability and applicability challenging in actual tunnelling environments.

The third category utilizes sensing signals and intelligent algorithms for damage identification. Pu Xiaobo employed vibration detection and machine learning for the diagnosis of abnormal tool damage [15]. Mohammad Amir Akhlaghi et al. identified the relationship between tool wear and acoustic/vibration signals using deep learning methods [16]. Agrawal Anil Kumar et al. used Multi-Layer Shallow Neural Networks (MSNN) to analyze the impact of thrust, torque, radial position, and rock properties (UCS and CAI) on wear [17]. Ghorbani Ebrahim established wear prediction models based on Gradient Boosting (GB) and Random Forest (RF) algorithms [18]. Akhlaghi A M adopted three machine learning methods with five-fold cross-validation to evaluate prediction accuracy for real-time wear estimation [19]. Linxuan Yuan selected 12 input parameters to facilitate rapid on-site assessment of tool wear [20]. Although mainstream deep learning models such as Long Short-Term Memory (LSTM) can automatically extract features, they rely on large-scale datasets and require long training times.

To achieve real-time continuous measurement, Fei Wang et al. optimized the coil geometry and circuit structure of an eddy current sensor using Ansoft Maxwell simulations [21]. Minsung Park et al. investigated the effects of various environments—including air, water, slurry, and silt—on sensor accuracy within shielded TBM chambers [22]. Eddy current sensors operate in harsh environments, such as those characterized by vibration and slag accumulation, which introduce substantial environmental noise into their signals. Furthermore, it is extremely difficult to obtain large, perfectly labelled real-world tool damage datasets, resulting in most samples for hob damage recognition being small-sample cases. With the advancement of deep learning, the Deep Forest (DF) model has emerged as a deep ensemble learning method based on tree models [23]. Through a multi-layer cascade structure, it achieves layer-by-layer feature enhancement, demonstrating excellent generalization and robustness under small samples, strong noise, and non-stationary signal conditions [24].

This paper achieves the identification and quantification of hob damage states by segmenting and resampling data on different types of hob damage, combined with the advantages of the DF model. For the classification of hob damage states, a comprehensive recognition accuracy of over 95% is regarded as meeting the requirements for engineering applications. For the quantitative evaluation of wear severity, a relative error of less than 10% is adopted as the engineering evaluation criterion, and a hob wear value of 15 mm is used as the tool replacement criterion. It is worth noting that the 15 mm threshold in this paper refers to the replacement criterion for hobs, which is an industry-recognized threshold based on empirical experience. In contrast, the industry-recognized replacement threshold for centre cutters and face cutters is 25 mm. The remainder of this paper is organized as follows: Section 2 describes the overall framework; Section 3 details the non-linear calibration of the eddy current sensor and wear volume; Section 4 presents the experimental system and dataset construction; and Section 5 analyzes the classification and regression results. The proposed method provides significant guidance for the intelligent condition monitoring of shield machines.

## 2. The Overall Framework of the Thesis

The operating environment of shield machines is highly variable and complex. Cutters in different spatial positions encounter distinct service conditions. Different damage types—such as normal wear, edge chipping, cracking, and chordal eccentric wear—correspond to specific operating conditions, constituting a classification problem. The severity of chipping directly determines cutter service life and replacement timing, representing a regression problem.

**Figure 1:**
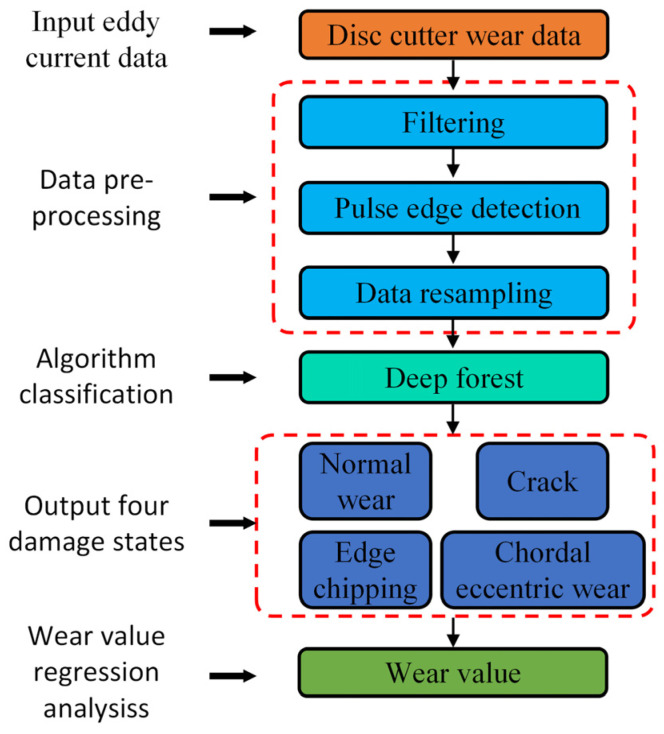
Roller cutter damage state identification process.

**Figure 2:**
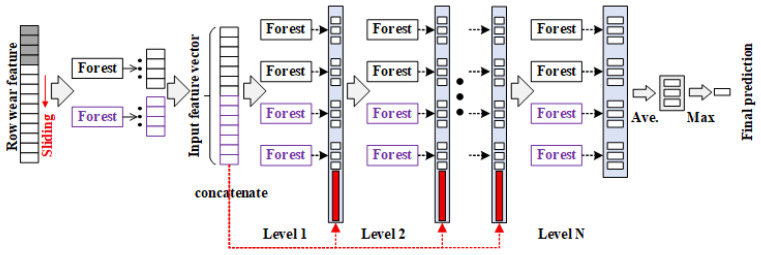
Theoretical framework of the deep forest model.

This paper proposes a disc cutter damage identification method based on eddy current sensing signals and a DF network. Figure 1 illustrates the flowchart of the identification process. First, a training dataset for various damage states is constructed by applying raw data processing techniques, including cutter ring data segmentation and damage feature extraction. Subsequently, the processed data are input into the DF ensemble learning model to achieve precise identification of four damage categories: normal, edge chipping, eccentric wear, and cracks. Building on this classification, regression analysis is further conducted to realize a quantitative assessment of the damage severity. The DF model is composed of multiple cascaded forest sub-modules. Each sub-module learns and extracts discriminative features layer by layer, and the final prediction is output through a cross-layer feature fusion mechanism. The overall architecture is shown in Figure 2.

## 3. Calibration of Eddy Current Sensors

**Figure 3:**
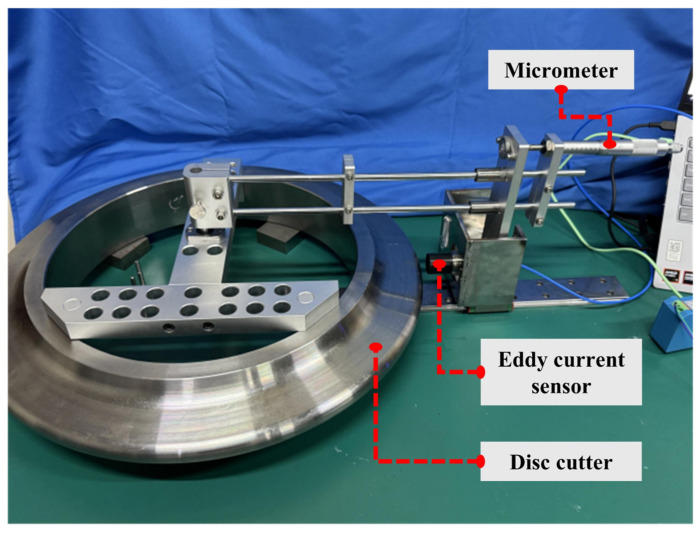
Eddy current sensor calibration platform.

Figure 3 illustrates the disc cutter wear calibration platform. The platform consists of a cutter ring, an eddy current sensor, a micrometer, and a linear guide. The cutter ring is fixed to a support base, while the probe of the eddy current sensor is aligned with the cutter axis and connected to the guide rail via a slider, allowing for longitudinal movement along the optical axis. The micrometer is fixed to the optical axis; rotating it adjusts the gap between the probe and the cutter ring to simulate varying degrees of cutter wear.

**Figure 4:**
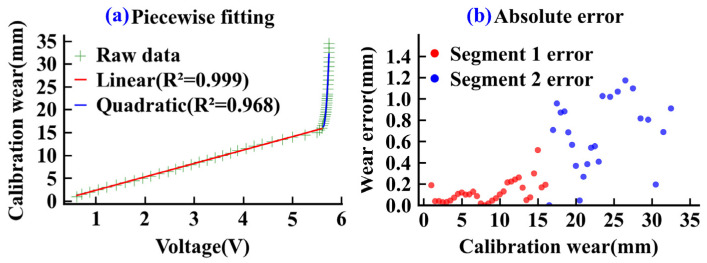
Eddy current sensor calibration. (**a**) Piecewise fitting, (**b**) Absolute error.

During the calibration process, the micrometer was used to adjust the gap, and the corresponding output voltages from the sensor were recorded to establish a mapping relationship between displacement and voltage for curve fitting. Figure 4 shows the calibration results and error analysis of the eddy current sensor. As shown in Figure 4a, the relationship between the output voltage and the calibrated wear volume varies across different intervals: it is approximately linear in the low-wear range, while it gradually exhibits non-linear characteristics in the larger wear range. Consequently, a piecewise fitting method was adopted to model the calibration curve, as expressed in Equations (1) and (2). Based on experimental data and the principle of error minimization, 5.6 V is determined as the optimal segmentation point, which corresponds to the transition region where the sensor sensitivity changes significantly. Figure 4b presents the absolute error results after piecewise fitting. The error is minimal in the low-wear interval and increases slightly in the high-wear interval, but overall, it satisfies the precision requirements for disc cutter wear measurement.

(1) $$W_{fit}=\left\{\begin{array}{cc} 2.96v-0.66 & v<5.6\;V\\ 1127v^{2}-12400v+34112.9 & v>5.6\;V \end{array}\right.$$

(2) $$\Delta W=|W_{cal}-W_{fit}|$$

In these equations, v represents the output voltage of the eddy current sensor, $W_{fit}$ is the predicted wear value calculated by the piecewise fitting model, $W_{cal}$ denotes the actual calibrated wear, and ΔW represents the absolute wear error.

## 4. Experimental System and Dataset Construction

### 4.1. Simulation System for Disc Cutter Damage

**Figure 5:**
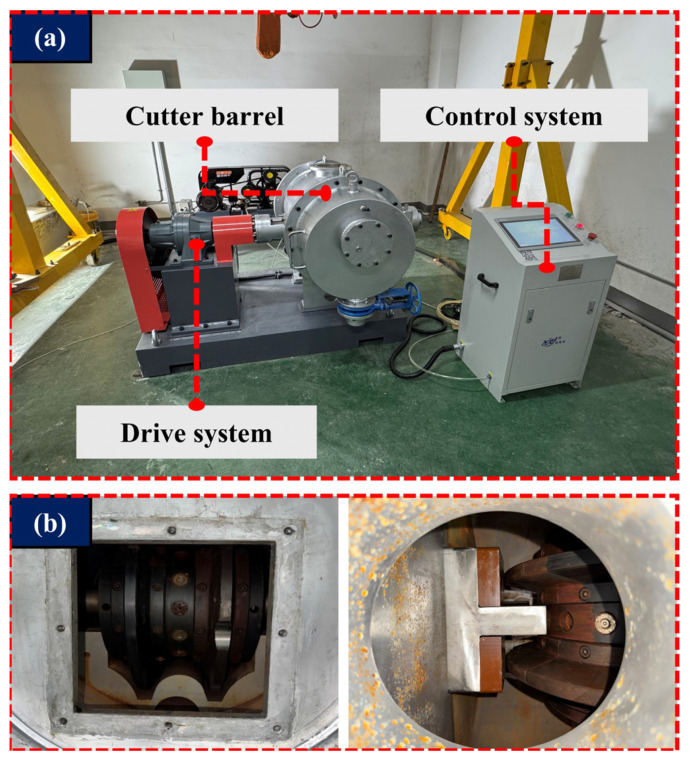
Hob damage measurement system. (**a**) Hob test bench, (**b**) internal structure of the hob barrel.

Figure 5 illustrates the experimental platform for disc cutter damage measurement. Figure 5a shows the primary components of the platform, including the drive system, the cutter barrel, and the control system. The simulated experimental test bench in this paper can reproduce hob rotation speeds ranging from 10 rpm to 70 rpm, stabilize the internal pressure of the cutter barrel up to 0.8 MPa, and replicate different muck conditions. Figure 5b presents a schematic diagram of the internal structure of the cutter barrel, where the cutter specimens are secured within the internal clamping mechanism. Due to the low sampling rate of on-site disc cutter wear data, it is not possible to accurately obtain the signal data of cutter damage. Therefore, this paper adopts a wear data acquisition system developed by our team, with a sampling rate of 200 Hz, which can accurately capture the signal characteristics of disc cutter damage in the field.

### 4.2. Construction of the Disc Cutter Damage Dataset

**Figure 6:**
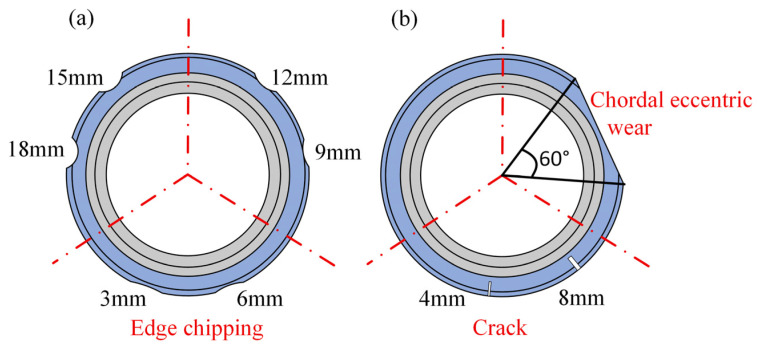
Hob cutter ring. (**a**) Hob cutter ring 1 with edge chipping, (**b**) hob cutter ring 2 with cracks and chordal eccentric wear.

Figure 6 illustrates the typical failure features manually machined on two cutter rings to construct the disc cutter damage dataset. The test disc cutter utilizes a split structure, consisting of three cutter rings spliced axially. Figure 6a shows six edge-chipping features circumferentially distributed on the same cutter ring, each with a width of 60 mm. Figure 6b displays composite damage features on another cutter ring, including two cracks with a depth of 25 mm and a chordal eccentric wear zone with a 60° angle. These designs provide representative samples for damage feature extraction and state identification models.

**Figure 7:**
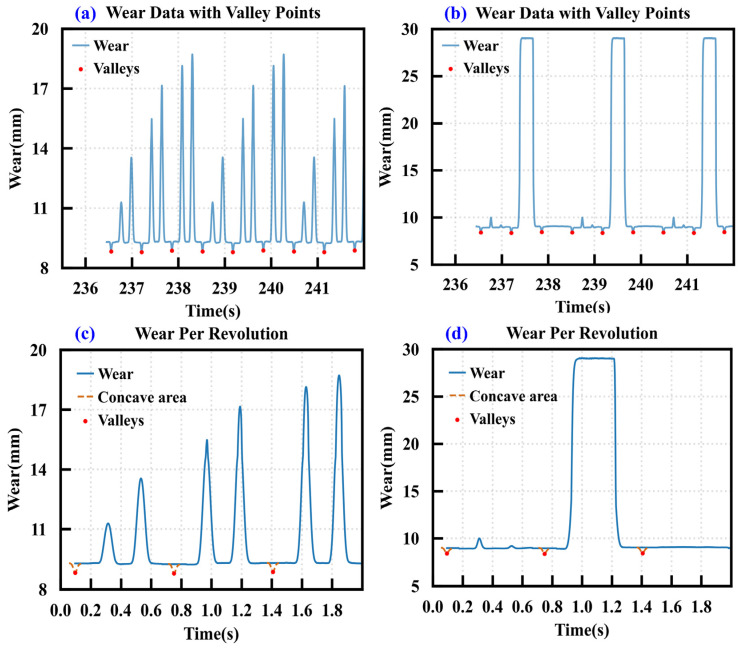
Extraction of single-ring wear data. (**a**) Multi-ring wear data of cutter ring 1, (**b**) multi-ring wear data of cutter ring 2, (**c**) single-ring wear data of cutter ring 1, (**d**) single-ring wear data of cutter ring 2.

Figure 7 demonstrates the extraction process and processing effects of the single-ring wear data. Figure 7a,b show the multi-ring wear signals collected during cutter rotation. Since the disc cutter is composed of three axially spliced rings, local depressions periodically appear in the signal at the junction areas. This anomaly stems from structural discontinuities rather than actual wear. To address this, a sliding window-based local minima detection method was employed to identify signal valleys. A window of length L slides along the time axis with a step size S to find local minimum values within each window as candidate points. After merging these points, a stable sequence of valleys is obtained. Subsequently, the anomalous segments are smoothed and corrected using these valleys as centres. Figure 7c,d display the single-ring wear data extracted using adjacent valleys as boundaries. The periodic depression interference has been effectively eliminated, providing a reliable data foundation for subsequent feature construction and model training.

**Figure 8:**
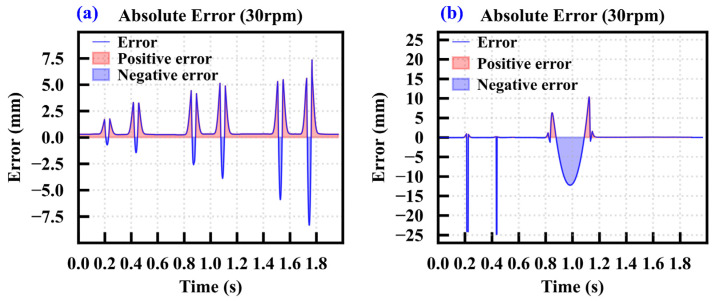
Absolute error. (**a**) Absolute error of cutter ring 1, (**b**) absolute error of cutter ring 2.

To quantitatively evaluate the reliability of the extracted single-ring wear values, an error analysis was performed, and the results are shown in Figure 8. The error ΔW is defined as the difference between the predicted wear value and the calibrated value, as expressed in Equation (3):

(3) $$\Delta W=W_{fit}-W_{act}$$

In this equation, W_fit_ represents the predicted wear value calculated by the piecewise fitting model, and W_act_ denotes the actual wear value of the disc cutter. From the overall trend, the error curve oscillates slightly around the zero value for most of the duration, indicating that the extraction method is stable and reliable in normal wear segments. In specific time intervals, the error curve exhibits significant positive and negative fluctuations, and the positions of these fluctuations align well with the damage locations on the cutter ring. For abrupt wear types such as edge chipping and cracks, the error curve typically presents narrow and sharp negative peaks. This is primarily due to the geometric discrepancies between the calibration process and the actual damage morphology.

**Figure 9:**
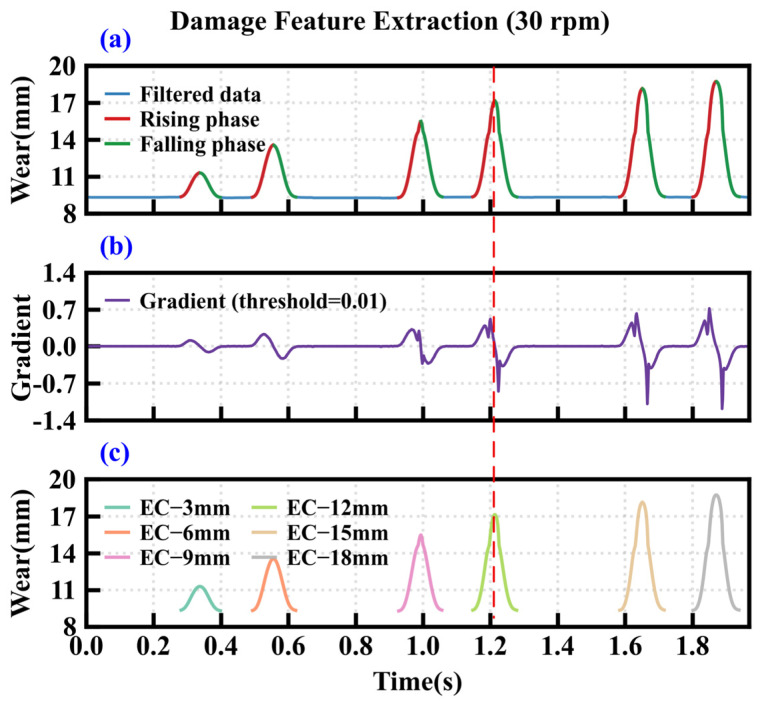
Damage feature extraction of cutter ring 1. (**a**) Filtered data, (**b**) first-order gradient, (**c**) damage feature.

**Figure 10:**
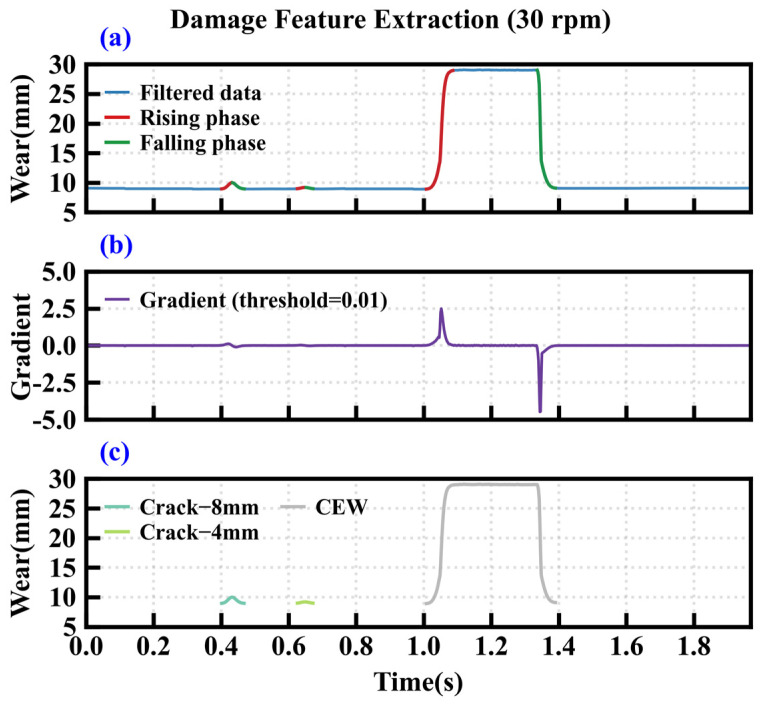
Damage feature extraction of cutter ring 2. (**a**) Filtered data, (**b**) first-order gradient, (**c**) damage feature.

Figure 9 demonstrates the extraction results of hob wear features under the operating condition of cutter ring 1. Figure 9a shows the raw time-domain data of the single-ring wear signal, while Figure 9b displays the corresponding first-order gradient signal. The specific start and end positions of the cutter damage are manifested as distinct positive and negative mutations in the gradient signal. By setting the positive gradient threshold to +0.01 V/s, the negative gradient threshold to −0.01 V/s, and the minimum duration constraint to 5 ms. Independent damage signals can be partitioned from the original signal. The final feature extraction results are shown in Figure 9c. Six edge-chipping features were successfully extracted from cutter ring 1. And chordal eccentric wear and crack features were extracted from cutter ring 2 in Figure 10. The significant differences in amplitude and duration among various damage states indicate that this method can effectively isolate and characterize typical disc cutter damage conditions.

## 5. Analysis of Model Prediction Results

### 5.1. Sample Data Preprocessing Process

**Figure 11:**
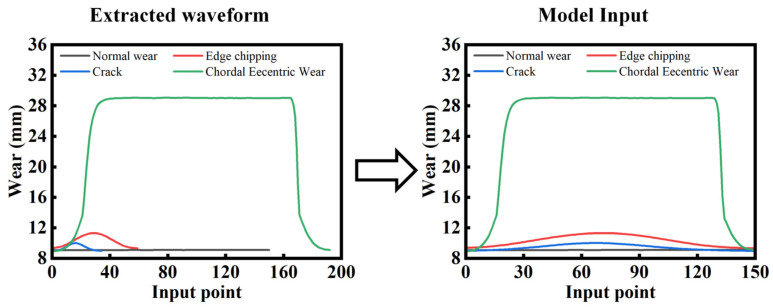
Data resampling for different types of injuries.

To ensure consistent data length across different injury types and maintain feature quality for proper model training, the paper resamples signals from various injuries. The resampled data are uniformly set to 150 data points in length, as shown in Figure 11.

During actual tunnelling operations, the probability of cutting edge chipping is significantly higher than that of tangential wear. The former is a common form of damage caused by fatigue or hard rock during the cutting process, while the latter is primarily attributed to roller bit jamming. Therefore, the data proportions presented in the paper align closely with real-world conditions.

### 5.2. Classification Prediction of Disc Cutter Damage States

#### 5.2.1. Training Parameters of the Deep Forest Classification Model

The DF model employed in this study utilizes a cascaded forest structure for layer-by-layer learning. Its workflow primarily consists of the following steps: First-layer training, where multiple random forest base learners are trained in parallel on the original input features. In this model, the first layer contains four different random forests, each consisting of 100 decision trees. Feature enhancement, where the class probability distribution output in each layer (i.e., the predicted probability vector for each sample) is concatenated with the original features to form a new augmented feature representation, which serves as the input for the next layer. Layer-by-layer training, where subsequent layers continue to train new sets of random forests based on the augmented features. This process proceeds sequentially, with each layer utilizing the probability features from the previous layer to learn richer representations. Automatic termination, where an early stopping mechanism is adopted during the training process. Training automatically terminates when the model’s performance on the validation set no longer improves significantly with additional layers or when the preset maximum number of layers (max_layers) is reached, thereby preventing overfitting and controlling computational costs. The various parameters of the model are shown in Table 1.

The meanings of the parameters in the table are as follows: n_e_ specifies the number of random forests used in each layer; n_tree_ denotes the number of decision trees within each random forest; l_max_ is the maximum number of layers in the cascade structure; n_jobs_ represents the parallel computing setting; and R_s_ is the seed for the random number generator to ensure reproducibility.

#### 5.2.2. Performance Evaluation of the Classification Model

**Figure 12:**
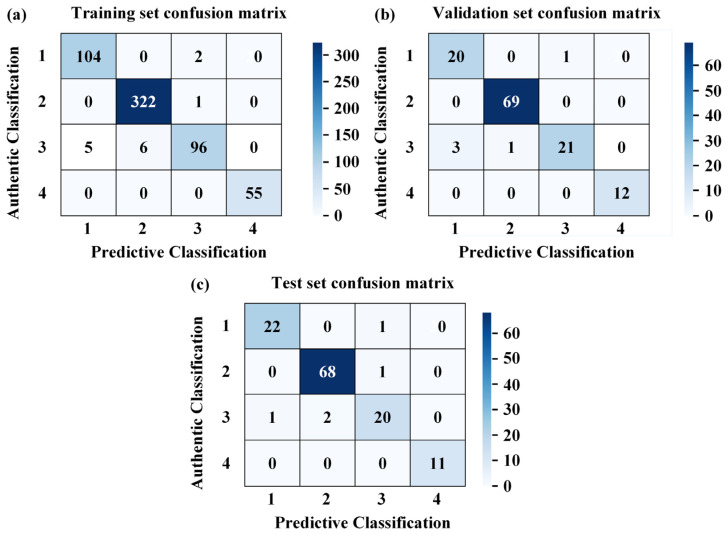
Confusion matrix of the dataset. (**a**) Training set confusion matrix, (**b**) verification set confusion matrix, (**c**) test set confusion matrix.

The typical damage forms of the cutting teeth of the shield machine include normal wear, edge chipping, cracks, and chordal eccentric wear, etc. The corresponding model classification numbers for this paper are shown in Table 2. The confusion matrices for different states were obtained through model training. As shown in Figure 12, the constructed model demonstrates exceptional classification performance across the training, validation, and test sets. The overall accuracy reached 98%, 96%, and 96%, respectively, with weighted average F1-scores exceeding 0.96 (refer to Table 3, Table 4 and Table 5). The minimal performance gap between the training and test sets (an accuracy difference of only 2%) indicates that the model possesses strong generalization capabilities, without significant overfitting or underfitting. The model can stably adapt to various data distribution scenarios, providing a reliable technical foundation for damage type identification tasks in structural health monitoring. The sample sizes for the training, validation, and test sets were 593, 126, and 127, respectively.

Training Set (591 samples): The model demonstrated balanced and efficient recognition performance across all categories in the training set: Chordal Eccentric Wear (Category 4, sample size: 55): Exhibited the best performance with precision, recall, and F1-score all reaching 1.00. This indicates that the features of this damage type are highly distinctive, allowing the model to achieve perfect identification. Chordal eccentric wear serves as a direct indicator of the actual operational status of the disc cutter. Although this feature is highly distinctive and easily distinguishable from other damage types in terms of data characteristics, manual identification becomes impractical in multi-cutter operation scenarios. Therefore, model-based automatic prediction of this wear pattern remains essential in practice. Edge Chipping (Category 2, sample size: 323): Ranked second with an F1-score of 0.99. The large sample size enabled the model to fully learn the features of this category, resulting in high identification stability. Normal Wear (Category 1, sample size: 106): Achieved an F1-score of 0.97, with a precision of 0.95 and a recall of 0.98. The recall was slightly higher than the precision, reflecting a low miss-detection rate for this category. Cracks (Category 3, sample size: 107): Showed relatively weaker performance with a recall of 0.90, which is lower than that of other categories. Potential reasons include the overlap of features with other damage types or the presence of hard-to-classify samples in the training set, leading to slightly insufficient identification completeness for this category.

Validation Set (127 samples): The validation set was used for hyperparameter tuning, and its performance reflects the model’s preliminary adaptability to unseen data: Chordal Eccentric Wear (sample size: 12): All metrics remained at 1.00, further confirming the uniqueness of these features and the robustness of the model’s recognition. Edge Chipping (sample size: 69): Achieved an F1-score of 0.99, sustaining the excellent performance seen in the training set and indicating good transferability of the model’s recognition capability for this category. Normal Wear (sample size: 21): The precision was 0.87 (the lowest in the entire dataset), the recall was 0.95, and the F1-score was 0.91. The decrease in precision may be attributed to the small sample size or specific anomalous samples in the validation set; however, the high recall indicates that the miss-detection rate remains low. Cracks (sample size: 25): The recall was 0.84, the lowest in the validation set, which is consistent with the training set performance. This confirms that this category remains the primary challenge for recognition due to the difficulty in feature differentiation.

Test Set (126 samples): The test set is independent of the training and validation processes, and its performance directly reflects the model’s potential for practical applications. Overall Accuracy: The overall accuracy (96%) was consistent with the validation set, demonstrating the model’s stable engineering applicability. Chordal Eccentric Wear (11 samples): Maintained a 100% recognition rate, proving once again that the model’s identification of this category is completely reliable. Normal Wear (23 samples) and Edge Chipping (69 samples): Both showed stable performance with F1-scores of 0.96 and 0.98, respectively. These results are slightly higher than those in the validation set, indicating that the model adapted well to these features in the test set. Cracks (23 samples): The F1-score was 0.89, with a precision of 0.91 and a recall of 0.87. Although these remain the lowest among all categories, the performance did not degrade, showing that the model’s recognition capability for this class remains consistent across different data distributions.

**Figure 13:**
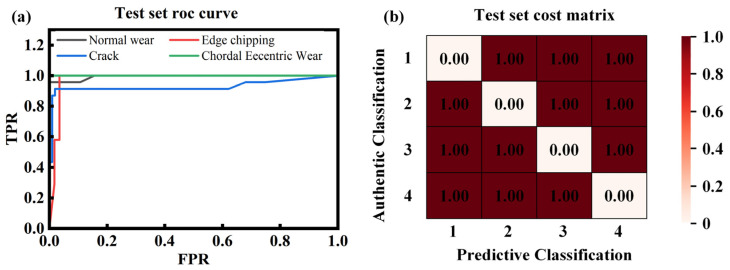
The ROC curve and cost matrix of the classification model’s test set. (**a**) Test set roc curve, (**b**) test set constant matrix.

This paper evaluated model performance using the Receiver Operating Characteristic (ROC) curve and the Area Under the Curve (AUC), as shown in Figure 13. The results indicate excellent performance, with macro-average and micro-average AUCs of 0.9772 and 0.9852, respectively. Category 4 achieved a perfect AUC of 1.0, and Class 1 was near-perfect at 0.9943. Categories 2 and 3 also performed strongly, with AUCs of 0.9776 and 0.9287. These findings demonstrate the model’s high classification capability, making it well-suited for equal-cost scenarios.

The model demonstrates excellent overall classification performance, with accuracies of at least 96% and weighted average F1-scores exceeding 0.96 across the training, validation, and test sets. These results satisfy the practical requirements for multi-type damage identification in structural health monitoring. Among the categories, chordal eccentric wear yielded the best recognition results due to its high feature distinctiveness. Edge chipping exhibited the highest identification stability, likely due to a sufficient sample size and thorough model learning. Cracks were identified as the weakest link in the model’s performance, consistently showing lower recall rates (0.84–0.90) and F1-scores (0.89–0.93) across all three datasets, indicating this is a key area for future improvement. The model also possesses strong generalization capabilities, as evidenced by the minimal performance gap between the training and test sets and the absence of overfitting, making it suitable for damage identification tasks in various scenarios.

Based on data and images, comparative analysis of signal characteristics across categories: Normal wear (Category 1) exhibits stable, low-fluctuation patterns with strong autocorrelation. Edge chipping (Category 2) features short-duration, high-amplitude impacts with concentrated energy. Cracks (Category 3) show weak, irregular fluctuations with small impact amplitudes. Chordal eccentric wear (Category 4) produces square-wave-like patterns with distinct characteristics. Crack propagation generates micro-impacts whose amplitudes resemble early-stage chipping, leading to misclassification between cracks and edge chipping. Late-stage normal wear generates intermittent high-frequency noise, which is misinterpreted as impact, leading to misclassification between normal wear and edge chipping.

#### 5.2.3. LSTM Model Classification Comparison

**Figure 14:**
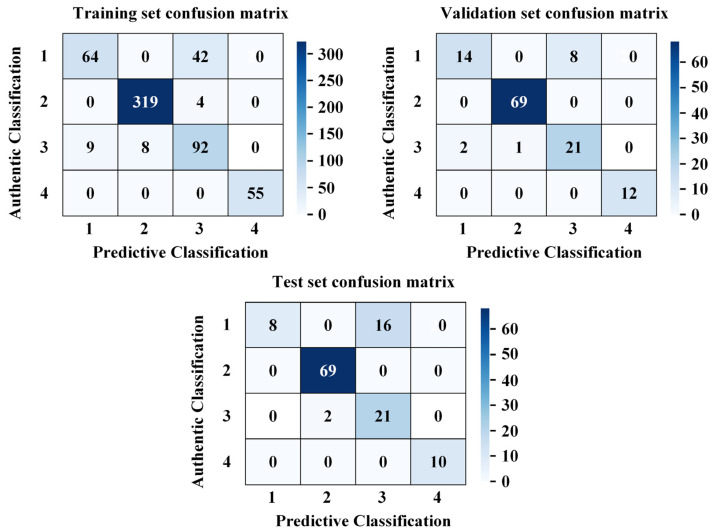
LSTM classification confusion matrix.

This study systematically compared the performance of Long Short-Term Memory (LSTM) networks and DF models in multi-class classification tasks using the same device and dataset, as shown in Figure 14. Experimental results demonstrate that the DF model significantly outperforms the LSTM model across all evaluation metrics, exhibiting superior classification accuracy, category balance, and generalization capabilities.

This network employs a two-layer LSTM architecture combined with dropout regularization and a dual fully connected layer structure. The parameters for each layer are 16,896, 0, 12,416, 0, 528, and 68, respectively, ultimately producing a 4-dimensional feature vector. The training set performance comparison between Deep Forest and LSTM is shown in Table 6.

This paper systematically compares the temporal overhead of DF models and LSTM networks when performing identical classification tasks. Experimental metrics encompass four dimensions: model training and evaluation on training/validation/test datasets. Results demonstrate that the DF model significantly outperforms LSTM in the preprocessing and training phases, reducing total time by 90.5%. Conversely, LSTM exhibits a slight advantage in single-run evaluation speed during validation and testing phases. This disparity stems from the fundamental architectural differences between the two models: DF leverages tree ensemble parallelization strategies, while LSTM relies on recurrent temporal computations and backpropagation. Table 7 and Table 8, respectively, present the hardware parameters for training the model, as well as the comparison of the training and validation times used by the DF and LSTM models on this platform.

### 5.3. Quantitative Prediction and Assessment of Disc Cutter Damage

#### 5.3.1. Parameters of the Quantitative Damage Regression Model

To evaluate the performance of the Deep Forest model in predicting target variables, systematic experiments were conducted in this study. The dataset was partitioned into training, validation, and test sets with a ratio of 69.8%: 15.0%: 15.2%, corresponding to sample sizes of 326, 70, and 71, respectively. This partitioning strategy aims to ensure that the model evaluation possesses strong generalization capabilities. The model adopts a two-layer cascade framework: the first layer consists of five base random forests (RF1–RF5), which expand the original 150-dimensional features to 155 dimensions; the second layer is a meta-random forest that performs the final prediction based on the augmented features. Through the collaboration of multi-level forests, this design is intended to capture complex patterns within the data more effectively. The relevant parameters of the multi-level model are shown in Table 9. D_max_ defines the maximum depth of a decision tree, which is the length of the longest path that can be generated within the tree.

#### 5.3.2. Analysis of Quantitative Regression Prediction Results

(1) Model Training and Prediction Results

The quantitative performance metrics for each dataset are presented in Table 10. The evaluation parameters, mean absolute error (MAE), mean square error (MSE), root mean square error (RMSE), and coefficient of determination (R^2^), are shown in Formulas (4)–(7). On the training set, the model demonstrates an exceptional fitting capability, with an MSE as low as 0.0033, an RMSE of 0.0578, and an R^2^ reaching 0.9999.

(4) $$\mathrm{MSE}=\frac{1}{n}\sum_{i=1}^{n}{\left(y_{i}-{\hat{y}}_{i}\right)}^{2}$$

(5) $$\mathrm{RMSE}=\sqrt{\frac{1}{n}\sum_{i=1}^{n}{\left(y_{i}-{\hat{y}}_{i}\right)}^{2}}$$

(6) $$R^{2}=1-\frac{\sum_{i=1}^{n}{\left(y_{i}-{\hat{y}}_{i}\right)}^{2}}{\sum_{i=1}^{n}{\left(y_{i}-\bar{y}\right)}^{2}}$$

(7) $$\mathrm{MAE}=\frac{1}{n}\sum_{i=1}^{n}|y_{i}-{\hat{y}}_{i}|$$

where $y_{i}$ represents the true value, ${\hat{y}}_{i}$ represents the predicted value, and $\bar{y}$ represents the mean of the true values.

These results indicate that the model has almost perfectly reconstructed the variations in the target variables in the training data. Although the performance on the validation and test sets is slightly lower than that on the training set, it remains at a high level: the validation set achieved an R^2^ of 0.9938 and an RMSE of 0.4217, while the test set yielded an R^2^ of 0.9940 and an RMSE of 0.4051. The high consistency of these metrics between the validation and test sets suggests that the model does not suffer from overfitting and possesses stable generalization performance.

**Figure 15:**
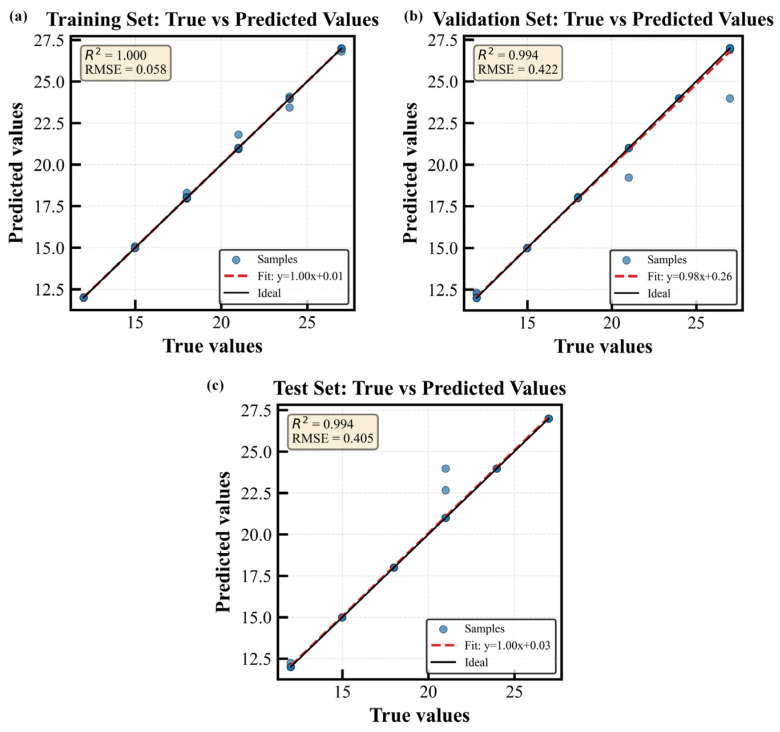
Regression curve of the dataset. (**a**) Training set regression curve, (**b**) validation set regression curve, (**c**) test set regression curve.

As shown in Figure 15, in the “Actual vs. Predicted” scatter plot for the training set, the regression line (slope is 1.000, intercept 0.01) almost perfectly coincides with the ideal line, with R^2^ of 1.000. The results for the validation and test sets further support the effectiveness of the model, as the sample points are closely distributed along the ideal line (e.g., the slope of the fitted line for the validation set is 0.98, and the intercept is 0.26).

(2) Feature Importance Analysis

**Figure 16:**
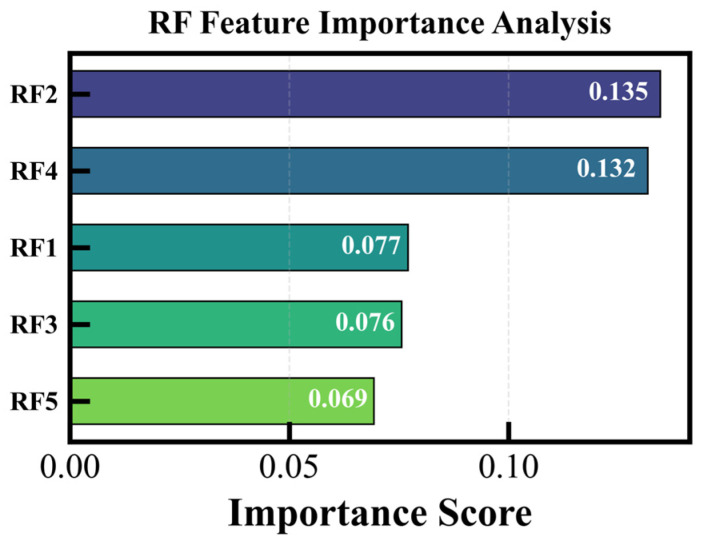
Importance of primordial forest characteristics.

Figure 16 illustrates the importance of primordial forest characteristics. The ranking of feature importance indicates that the augmented features from the second-layer meta-forest play a crucial role in the model, with a cumulative importance of 48.84%, nearly half of the total. In contrast, the combined contribution of the remaining 150 original features is 51.16%. This demonstrates that while the model enhances features, it also retains vital information from the original data structure, achieving an effective integration of abstract and primordial features. Within the RF feature group, RF2 and RF4 exhibit the highest importance, with a combined contribution of 26.64%, accounting for 54.5% of the RF group’s internal importance. Although RF5 has the lowest importance within the group (0.0693), it remains higher than most non-RF core features.

#### 5.3.3. Regression Model Ablation Experiment Comparison

To further compare the rationality of the selected parameters in the paper model, we conducted ablation experiments to verify the impact of the number of layers on the prediction performance, as shown in Table 11. Experimental results show that training time increases approximately linearly with network depth, as expected. The MSE, RMSE, and R^2^ show no significant differences in the prediction results across various layer counts. However, MAE drops significantly at two layers and shows limited improvement thereafter. Considering both accuracy and efficiency, the two-layer model achieves the lowest MAE with accuracy comparable to other configurations, while requiring substantially less training time than deeper models. Given the diminishing returns and potential overfitting risk beyond two layers, the two-layer model offers the best trade-off between performance and computational cost.

## 6. Conclusions

This study develops an intelligent identification and quantitative assessment framework for shield disc cutter damage based on the DF model. By employing a piecewise fitting method to calibrate the non-linear relationship between eddy current sensor signals and wear volume, a reliable data foundation for precise quantification was established, achieving high fitting coefficients in both linear and non-linear intervals. Through edge-based data segmentation and DF model training, the proposed classification model achieves an accuracy of 96% and a weighted average F1-score exceeding 0.96 across training, validation, and test sets. Furthermore, the two-layer cascaded regression model realizes high-precision continuous prediction of damage magnitude, yielding an R^2^ of 0.9940 and an RMSE of 0.4051. The integration of qualitative state identification and quantitative damage assessment enables accurate detection of edge chipping, cracks, and eccentric wear, and predicts chipping size. This methodology provides a scientific basis for optimizing cutter maintenance strategies, thereby reducing unplanned downtime and ensuring the safety and efficiency of shield tunnelling operations.

Although the test results in the paper were obtained under the condition of a broken hob in the laboratory, they still have certain guiding significance for the application in engineering fields. In future research, we will focus on the study of composite damage and will continuously collect on-site data for model updates.

## Figures and Tables

**Table 1:** Deep forest model training parameters.

n_e_	n_tree_	l_max_	n_jobs_	R_s_
4	100	20	−1	42

**Table 2:** State classification and selected sample data.

Normal Wear	Edge Chipping	Crack	Chordal Eccentric Wear
category 1	category 2	category 3	category 4

**Table 3:** Training set classification report.

Classification	Precision	Recall	F1-Score	Support
Category 1	0.95	0.98	0.97	106
Category 2	0.98	1.00	0.99	323
Category 3	0.97	0.90	0.93	107
Category 4	1.00	1.00	1.00	55

**Table 4:** Validation set classification report.

Classification	Precision	Recall	F1-Score	Support
Category 1	0.87	0.95	0.91	21
Category 2	0.99	1.00	0.99	69
Category 3	0.95	0.84	0.89	25
Category 4	1.00	1.00	1.00	12

**Table 5:** Test set classification report.

Classification	Precision	Recall	F1-Score	Support
Category 1	0.96	0.96	0.96	23
Category 2	0.97	0.99	0.98	69
Category 3	0.91	0.87	0.89	23
Category 4	1.00	1.00	1.00	11

**Table 6:** Performance comparison between Deep Forest and LSTM.

Name	Accuracy	F1-Score	F1-Score
Deep Forest	0.98	0.97	0.98
LSTM	0.89	0.86	0.89
Enhancement	10.11%	12.79%	10.11%

**Table 7:** Computer hardware for training models.

Memory	Processor
8 GB	Intel(R) Core (TM) i5-10500 CPU @ 3.10 GHz

**Table 8:** Time consumption for each component of the DF and LSTM classification models.

Name	Model Training	Training Set	Validation Set	Test Set
DF	3.4278 s	0.2622 s	0.2493 s	0.2515 s
LSTM	56.0792 s	0.3535 s	0.1204 s	0.1305 s

**Table 9:** Parameters at each level of the regression model.

Floor	n_e_	R_s_	D_max_
Floor 1	50	42	5
Floor 2	100	42	10

**Table 10:** Performance metrics for each dataset.

Metric	Training Set	Validation Set	Test Set
MSE	0.0033	0.1778	0.1641
MAE	0.0071	0.0800	0.0686
RMSE	0.0578	0.4217	0.4051
R^2^	0.9999	0.9938	0.9940

**Table 11:** Comparison of ablation test results on the test set of the regression model.

Layers	Time (s)	MSE	MAE	RMSE	R^2^
1	1.1320	0.1601	0.1666	0.4001	0.9942
2	3.6253	0.1641	0.0686	0.4051	0.9940
3	6.0080	0.1615	0.0708	0.4019	0.9941
4	8.2809	0.1603	0.0687	0.4004	0.9942
5	10.5668	0.1666	0.0686	0.4082	0.9939

## Data Availability

Data are contained within the article.
